# Estimated County-Level Prevalence of Selected Underlying Medical Conditions Associated with Increased Risk for Severe COVID-19 Illness — United States, 2018

**DOI:** 10.15585/mmwr.mm6929a1

**Published:** 2020-07-24

**Authors:** Hilda Razzaghi, Yan Wang, Hua Lu, Katherine E. Marshall, Nicole F. Dowling, Gabriela Paz-Bailey, Evelyn R. Twentyman, Georgina Peacock, Kurt J. Greenlund

**Affiliations:** ^1^CDC COVID-19 Response Team; ^2^Division of Population Health, National Center for Chronic Disease Prevention and Health Promotion, CDC.

Risk for severe coronavirus disease 2019 (COVID-19)–associated illness (illness requiring hospitalization, intensive care unit [ICU] admission, mechanical ventilation, or resulting in death) increases with increasing age as well as presence of underlying medical conditions that have shown strong and consistent evidence, including chronic obstructive pulmonary disease, cardiovascular disease, diabetes, chronic kidney disease, and obesity (*1*–*4*). Identifying and describing the prevalence of these conditions at the local level can help guide decision-making and efforts to prevent or control severe COVID-19–associated illness. Below state-level estimates, there is a lack of standardized publicly available data on underlying medical conditions that increase the risk for severe COVID-19–associated illness. A small area estimation approach was used to estimate county-level prevalence of selected conditions associated with severe COVID-19 disease among U.S. adults aged ≥18 years (*5*,*6*) using self-reported data from the 2018 Behavioral Risk Factor Surveillance System (BRFSS) and U.S. Census population data. The median prevalence of any underlying medical condition in residents among 3,142 counties in all 50 states and the District of Columbia (DC) was 47.2% (range = 22.0%–66.2%); counties with the highest prevalence were concentrated in the Southeast and Appalachian region. Whereas the estimated number of persons with any underlying medical condition was higher in population-dense metropolitan areas, overall prevalence was higher in rural nonmetropolitan areas. These data can provide important local-level information about the estimated number and proportion of persons with certain underlying medical conditions to help guide decisions regarding additional resource investment, and mitigation and prevention measures to slow the spread of COVID-19.

BRFSS is an annual, random-digit–dialed landline and mobile telephone survey of noninstitutionalized U.S. adults aged ≥18 years in all 50 states, DC, and U.S. territories. BRFSS collects self-reported information on selected health behaviors and conditions. Overall, 437,500 persons participated in the 2018 BRFSS survey, with a median weighted response rate of 49.9%.*

The underlying medical conditions included in these prevalence estimates were selected using the subset of the list of conditions with the strongest and most consistent evidence^†^ of association with higher risk for severe COVID-19–associated illness on CDC’s website as of June 25, 2020 (*2*) and for which questions on the BRFSS aligned. These included chronic obstructive pulmonary disease (COPD), heart conditions, diabetes mellitus, chronic kidney disease (CKD), and obesity (defined as body mass index [BMI] of ≥30 kg per m^2^). Conditions from the list of those with mixed and limited evidence^§^ of association with increased risk for severe COVID-19 illness were not included (*2*). An analysis of U.S. COVID-19 patient surveillance data found that hospitalizations were six times higher, ICU admissions five times higher, and deaths 12 times higher among patients with underlying medical conditions, compared with those without (*4*); however, that analysis included a narrower definition of obesity (BMI ≥40 kg per m^2^), and some, but not all conditions in both the strongest and most consistent evidence and mixed and limited evidence lists.

BRFSS respondents were classified as having an underlying medical condition if they answered “yes” to any of the following questions: “Have you ever been told by a doctor, nurse, or other health professional that you have COPD, emphysema, or chronic bronchitis; heart disease (angina or coronary heart disease, heart attack, or myocardial infarction); diabetes; or chronic kidney disease?” Respondent-reported height and weight were used to calculate BMI; respondents with BMI ≥30 kg per m^2^ were considered to have obesity. A created variable captured persons having any of these conditions.

Nationwide estimates of underlying medical conditions were weighted to adjust for survey design. For county-level prevalence, estimates of each and of any condition were generated using a multilevel regression and poststratification approach (*5*) for 3,142 counties in all 50 states and DC. This approach has been validated in comparison with direct BRFSS survey estimates and local surveys for multiple chronic disease measures at state and county levels (*5*,*6*). Briefly, a multilevel regression model was constructed for each outcome using individual-level age,^¶^ gender, race/ethnicity,** and educational-level^††^ data from the 2018 BRFSS, and data on county-level percentage of the adult population living at <150% of the poverty level from the 2014–2018 American Community Survey (ACS), a survey sent to about 3.5 million addresses each month that asks about topics not included on the decennial census, including education and employment. The model parameters were applied to 2018 Census county-level population estimates by age, gender, and race/ethnicity to calculate the predicted probability of each outcome. Because the U.S. Census Bureau does not provide county-level population data for education level by age, sex, and race/ethnicity, a bootstrapping approach^§§^ was used to impute it. The estimated prevalence was obtained by multiplying the probability by the total population by county. Model-based estimates for any condition were validated by comparing them with the weighted direct survey estimates from counties with sample size ≥500 (213) in BRFSS; the Pearson correlation coefficient was 0.89. The county-level estimates of having any underlying medical condition were categorized into six county urban/rural classifications using CDC’s National Center for Health Statistics definitions (large central metro/city, large fringe metro/suburb, medium metro, small metro, micropolitan, noncore/rural) (*7*). The overall weighted direct survey estimates were conducted using SUDAAN (version 11; RTI International), and other analyses were conducted using SAS (version 9.4; SAS Institute).

The nationwide prevalence of any of the five underlying medical conditions among adults aged ≥18 years was 40.7% (95% confidence interval [CI] = 40.4%–41.0%) (Table 1). The overall weighted prevalences of these conditions were 30.9% (obesity), 11.4% (diabetes), 6.9% (COPD), 6.8% (heart disease), and 3.1% (CKD).

**TABLE 1:** Nationwide and model-based county-level (n = 3,142) estimates of prevalence and number of adults aged ≥18 years with selected underlying medical conditions that might increase risk for severe COVID-19–associated illness — United States, 2018

Selected underlying medical condition*	Nationwide prevalence^†^ % (95% CI)	Median county prevalence^§^ % (range)	Median county no. of adults^†^ (range)
Any	40.7 (40.4, 41.0)	47.2 (22.0–66.2)	9,743 (41–2,877,316)
Obesity (BMI ≥30 kg/m^2^)	30.9 (30.6, 31.2)	35.4 (15.2– 49.9)	7,174 (25–2,097,906)
Diabetes mellitus	11.4 (11.2, 11.6)	12.8 (6.1–25.6)	2,742 (11–952,335)
COPD	6.9 (6.7, 7.0)	8.9 (3.5–19.9)	1,962 (7–434, 075)
Heart disease	6.8 (6.7, 7.0)	8.6 (3.5–15.1)	1, 811 (7–434,790)
Chronic kidney disease	3.1 (3.0, 3.3)	3.4 (1.8–6.2)	717 (3–237,766)

**Abbreviations:** BMI = body mass index; CI = confidence interval; COPD = chronic obstructive pulmonary disease; COVID-19 = coronavirus disease 2019.

* Diabetes mellitus includes both type 1 and type 2 diabetes. COPD includes emphysema and chronic bronchitis. Heart disease includes angina or coronary heart disease, and heart attack or myocardial infarction.

^†^ Weighted direct estimates from the Behavioral Risk Factor Surveillance System, 2018.

^§^ Prevalence and number of adults estimated for 3,142 counties using a multilevel regression and poststratification approach applied to 2018 Behavioral Risk Factor Surveillance System data.

Among 3,142 counties, the median estimated (modeled) county prevalence of any underlying medical condition was 47.2% (range = 22.0%–66.2%); obesity, 35.4% (range = 15.2%–49.9%); diabetes, 12.8% (range = 6.1%–25.6%); COPD, 8.9% (range = 3.5%–19.9%); heart disease, 8.6% (range = 3.5%–15.1%); and CKD, 3.4% (range = 1.8%–6.2%) (Table 1).

Counties with the highest prevalences of any condition were concentrated in Southeastern states, particularly in Alabama, Arkansas, Kentucky, Louisiana, Mississippi, Tennessee, and West Virginia, as well as some counties in Oklahoma, South Dakota, Texas, and northern Michigan, among others (Figure) (Supplementary Table, https://stacks.cdc.gov/view/cdc/90519). The estimated number of adults with any condition generally followed the population distribution, with higher estimated numbers of persons with any underlying medical conditions in more highly populated areas.

**FIGURE:**
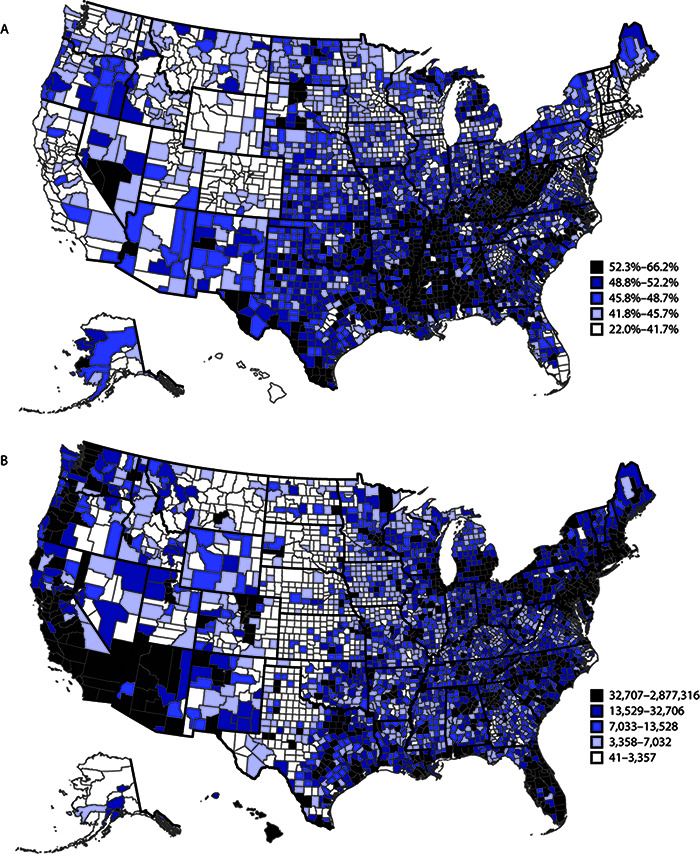
Model-based estimates of U.S. prevalence (A) and number (B) of adults aged ≥18 years with any selected underlying medical condition,* by county — United States, 2018 * Selected underlying conditions include chronic obstructive pulmonary disease, emphysema, or chronic bronchitis; heart disease (angina or coronary heart disease, heart attack, or myocardial infarction); diabetes; chronic kidney disease; or obesity (body mass index ≥30 kg/m^2^).

The estimated median prevalence of any condition generally increased with increasing rurality, ranging from 39.4% in large central metro counties to 48.8% in noncore counties (Table 2); the estimated median number of persons with any underlying condition ranged from 4,300 in noncore counties to 301,744 in large central metro counties.

**TABLE 2:** Model-based estimates of prevalence and number of persons aged ≥18 years with any select underlying medical condition, by urban/rural county classification — United States, 2018

County classification*	No. of counties	Median county prevalence % (range)	Median county no. of persons (range)
**Metropolitan**			
Large central metro^†^	68	39.4 (23.9–48.1)	301,744 (43,770–2,877,316)
Large fringe metro^§^	368	43.9 (26.4–56.9)	34,221 (1,611–725,284)
Medium metro^¶^	372	45.5 (22.0–61.7)	33,687 (659–332,209)
Small metro**	358	45.8 (27.8–62.2)	26,683 (41–87,153)
**Nonmetropolitan**			
Micropolitan^††^	641	47.8 (24.3–64.6)	13,979 (176–59,820)
Noncore^§§^	1,335	48.8 (26.8–66.2)	4,300 (47–29,469)

* Based on 2013 Urban-Rural Classification Scheme for Counties from the National Center for Health Statistics, CDC.

^†^ Large central metro counties in metropolitan statistical areas (MSAs) of 1 million population that 1) contain the entire population of the largest principal city of the MSA, or 2) are completely contained within the largest principal city of the MSA, or 3) contain ≥250,000 residents of any principal city in the MSA.

^§^ Large fringe metro counties in MSA of ≥1 million population that do not qualify as large central.

^¶^ Medium metro counties in MSA of 250,000–999,999 population.

** Small metro counties are counties in MSAs of <250,000 population.

^††^ Micropolitan counties in MSAs.

^§§^ Noncore counties not in MSAs.

## Discussion

Three recent studies have reported that underlying medical conditions are highly prevalent among U.S. COVID-19 patients requiring hospitalization and ICU admission (*3*,*4*,*8*). In this report, the median county prevalence of any of five underlying medical conditions that increase the risk for severe COVID-19–associated illness was 47.2%, and prevalences were higher in counties in the southeastern United States and in more rural counties. These county level estimates can be used together with data on hospitalizations, ICU admissions, and ventilator use among COVID-19 patients with underlying conditions when planning for mitigation efforts and additional resource investment, including hospital beds, staffing, ventilators, and other medical supplies that might be needed to treat persons with underlying medical conditions, should they become ill with COVID-19.

The percentage of the population (prevalence) and the estimated numbers of adults with underlying medical conditions provide information for planning and have implications for health care resource utilization. Areas with comparatively lower prevalences but large populations, such as metropolitan areas, might still have large numbers of persons with underlying medical conditions at increased risk for severe COVID-19 illness. Conversely, areas with smaller populations but a comparatively higher prevalence of persons with underlying medical conditions might also have substantial need for additional resources to treat severe COVID-19 illness. Health care in rural counties is often underresourced,^¶¶^ and rural communities might have limited access to adequate care, which could further increase risk for poor COVID-19–associated outcomes. Prevalence estimates help highlight counties with a higher relative need for resources, whereas estimates of numbers of persons with underlying medical conditions help identify overall need by county; both can help decision-makers predict resource needs and develop resource allocation plans.

The findings in this report are subject to at least five limitations. First, estimates were based on BRFSS data and subject to survey biases such as nonresponse, social desirability, and recall and knowledge of having a particular condition. Second, BRFSS data do not include all underlying medical conditions that might increase risk for severe COVID-19 illness, such as sickle cell disease, or information on organ transplant or disease severity. Third, some of the underlying medical conditions included in BRFSS might not exactly capture those conditions with the strongest and most consistent evidence such as specific heart conditions (e.g., cardiomyopathies and heart failure) or specific type of diabetes. Further, because COVID-19 is a novel disease and information regarding risk factors for severe illness is evolving, additional underlying medical conditions might be added in the future (as an example, cancer was added to the list after these analyses were conducted). Fourth, BRFSS data are collected for noninstitutionalized civilian persons and exclude populations that might be particularly vulnerable to severe COVID-19 illness, including those living in long-term care facilities and incarcerated populations, and might therefore not be representative for those groups. Finally, these estimates might be imprecise because of the multilevel regression modeling process and county-level population estimation. 

These findings can be used by state and local decision-makers to help identify areas at higher risk for severe COVID-19–associated illness because of underlying medical conditions and guide resource allocation and implementation of prevention and mitigation strategies. Future analyses could include weighting the contribution of each underlying medical condition according to the risk for severe COVID-19–associated outcomes, as well as identifying and incorporating other aspects of vulnerability to both infection and severe outcomes to better estimate the number of persons at increased risk for COVID-19. These findings highlight the prevalence of underlying medical conditions at the local (county) level that are important causes of morbidity and mortality on their own and increase risk for severe COVID-19–associated illness. These findings also emphasize the importance of prevention efforts to reduce the prevalence of these underlying medical conditions and their risk factors such as smoking, unhealthy diet, and lack of physical activity.

**Summary**

**What is already known about this topic?**

Older adults and those with chronic obstructive pulmonary disease, heart disease, diabetes, chronic kidney disease, and obesity are at higher risk for severe COVID-19–associated illness.

**What is added by this report?**

The median model-based estimate of the prevalence of any of five underlying medical conditions associated with increased risk for severe COVID-19–associated illness among U.S. adults was 47.2% among 3,142 U.S. counties. The estimated number of persons with these conditions followed population distributions, but prevalence was higher in more rural counties.

**What are the implications for public health practice?**

The findings can help local decision-makers identify areas at higher risk for severe COVID-19 illness in their jurisdictions and guide resource allocation and implementation of community mitigation strategies.

## Footnotes

*
https://www.cdc.gov/brfss/index.html .

† Conditions with consistent evidence of increased risk for severe COVID-19–associated illness from multiple small studies or a strong association from a large study.

§ Conditions for which multiple studies have reached different conclusions about risk associated with that condition. Those with limited evidence are those for which consistent evidence has been reported from a small number of studies. https://www.cdc.gov/coronavirus/2019-ncov/need-extra-precautions/evidence-table.html.

¶ Age was categorized into 13 age groups at 5-year intervals for ages ≥18 years.

** Race/ethnicity was categorized as non-Hispanic white, non-Hispanic African American, non-Hispanic American Indian or Alaska Native, non-Hispanic Asian, non-Hispanic Native Hawaiian/other Pacific Islander, other single non-Hispanic race, two or more non-Hispanic race groups, and Hispanic.

†† Education was categorized as less than high school, high school graduate, some college or technical school, or college graduate.

§§ https://ww2.amstat.org/meetings/jsm/2016/onlineprogram/AbstractDetails.cfm?abstractid=319359.

¶¶
https://www.aha.org/system/files/2019-02/rural-report-2019.pdf .
